# A Microporous Zn(bdc)(ted)_0.5_ with Super High Ethane Uptake for Efficient Selective Adsorption and Separation of Light Hydrocarbons

**DOI:** 10.3390/molecules28166000

**Published:** 2023-08-10

**Authors:** Feng Xu, Yilu Wu, Juan Wu, Daofei Lv, Jian Yan, Xun Wang, Xin Chen, Zewei Liu, Junjie Peng

**Affiliations:** 1 School of Environment and Chemical Engineering, Foshan University, Foshan 528000, China; fengxu@fosu.edu.cn (F.X.); ceyiluwu@foxmail.com (Y.W.); lvdaofei@163.com (D.L.); yanjian@fosu.edu.cn (J.Y.); cexunwang@fosu.edu.cn (X.W.); chenxin@fosu.edu.cn (X.C.); 2 College of Environmental Monitoring, Guangdong Polyytechnic of Environmetal Protection Engineering, Foshan 528216, China; wjqwer0059@163.com

**Keywords:** MOFs, light hydrocarbons, adsorption and separation, Zn(bdc)(ted)_0.5_

## Abstract

Separating light hydrocarbons (C_2_H_6_, C_3_H_8_, and C_4_H_10_) from CH_4_ is challenging but important for natural gas upgrading. A microporous metal-organic framework, Zn(bdc)(ted)_0.5_, based on terephthalic acid (bdc) and 1,4-diazabicyclo[2.2.2]octane (ted) ligands, is synthesized and characterized through various techniques, including powder X-ray diffraction (PXRD), scanning electron microscopy (SEM), thermogravimetric analysis (TGA), and porosity analysis. The adsorption isotherms of light hydrocarbons on the material are measured and the isosteric adsorption heats of CH_4_, C_2_H_6_, C_3_H_8_, and C_4_H_10_ are calculated. The prediction of C2–4/C1 adsorption selectivities is accomplished using ideal adsorbed solution theory (IAST). The results indicate that the material exhibits exceptional characteristics, including a Brunauer-Emmett-Teller (BET) surface area of 1904 m^2^/g and a pore volume of 0.73 cm^3^/g. Notably, the material demonstrates remarkable C_2_H_6_ adsorption capacities (4.9 mmol/g), while CH_4_ uptake remains minimal at 0.4 mmol/g at 298 K and 100 kPa. These findings surpass those of most reported MOFs, highlighting the material’s outstanding performance. The isosteric adsorption heats of C_2_H_6_, C_3_H_8_, and C_4_H_10_ on the Zn(bdc)(ted)_0.5_ are higher than CH_4_, suggesting a stronger interaction between C_2_H_6_, C_3_H_8_, and C_4_H_10_ molecules and Zn(bdc)(ted)_0.5_. The molecular simulation reveals that Zn(bdc)(ted)_0.5_ prefers to adsorb hydrocarbon molecules with richer C-H bonds and larger polarizability, which results in a stronger dispersion force generated by an adsorbent-adsorbate induced polarization effect. Therefore, the selectivity of C_4_H_10_/CH_4_ is up to 180 at 100 kPa, C_3_H_8_/CH_4_ selectivity is 67, and the selectivity of C_2_H_6_/CH_4_ is 13, showing a great potential for separating C2–4 over methane.

## 1. Introduction

One of the most pressing environmental challenges is the ongoing rise in atmospheric carbon dioxide resulting from extensive fossil fuel consumption. In comparison to traditional fossil fuels, natural gas exhibits diminished carbon emissions, relatively lower pollutant discharges (such as NOx), and can be harnessed to mitigate fluctuations in renewable energy sources. Natural gas, primarily composed of methane (75–90%), also encompasses impurities (such as C_2_H_6_, C_3_H_8_, and C_4_H_10_, collectively ranging from 0–20%) [1,2]. Hence, the extraction of heavier hydrocarbons becomes imperative to meet the requisites of natural gas enhancement and secure storage. It is challenging to effectively separate natural gas into a single component, but it is very important in the petrochemical industry. Innovative methods for the efficient separation of methane from mixed light hydrocarbons into pure components must be investigated, as traditional approaches (such as cryogenic distillation) are very energy intensive [3]. Recent advancements have highlighted the significance of adsorptive separation technology as an efficient, cost-effective, and low-energy-consumption technique. In the process of adsorption, the meticulous selection of a suitable adsorbent plays a crucial role.

In the context of porous materials, metal-organic frameworks (MOFs) are the focus of significant attention. MOFs are being considered for their potential application in numerous areas, ranging from adsorption and separation [4,5,6] to catalysis [7,8], luminescence [9,10,11], electronics [12,13], magnetism [14], drug delivery [15], and sensing [16]. To date, MOF materials have been developed into prospective resources for methane purification. Yuan et al. [17] reported a facile synthesis of MIL-100 (Fe) at room temperature, which exhibited CH_4_, C_2_H_6_, and C_3_H_8_ uptake capacities of 0.36, 2.22, and 6.78 mmol/g, respectively, at 100 kPa and 298 K. The IAST-predicted selectivity was 6.0 for C_2_H_6_/CH_4_ at 298 K. Lv et al. [18] found that CTGU-15 demonstrated exceptionally high C_3_H_8_ uptake (12.13 mmol/g) and relatively low CH_4_ uptake (0.40 mmol/g) at 298 K and 100 kPa, but it had low C_2_H_6_ uptake (2.13 mmol/g) and low C_2_H_6_/CH_4_ selectivity (5.2). Chen et al. [19] discovered that InOF-1 exhibited a high C_2_H_6_/CH_4_ selectivity of 17 at 100 kPa and 298 K. However, it also exhibited a relatively high CH_4_ uptake of 0.64 mmol/g. Shi et al. [20] reported that PCN-224 demonstrated a high C_2_H_6_/CH_4_ selectivity of 12 at 100 kPa and 298 K. Nevertheless, this material exhibited a relatively low C_2_H_6_ uptake of 2.94 mmol/g. Therefore, the development of MOFs that exhibit low CH_4_ uptake, high C_2_H_6_ uptakes, and high C_2_H_6_/CH_4_ selectivities is of utmost importance to meet the industrial application requirements for natural gas purification.

This study presents a comprehensive analysis of a highly microporous Zn-based MOF, Zn(bdc)(ted)_0.5_. The main focus is investigating the adsorption behavior of light hydrocarbons, including CH_4_, C_2_H_6_, C_3_H_8_, and C_4_H_10_. The material is thoroughly characterized using X-ray diffraction (XRD), thermogravimetric analysis (TGA), and N_2_ adsorption techniques. To examine the adsorption isotherms of light hydrocarbons (CH_4_, C_2_H_6_, C_3_H_8_, and C_4_H_10_) at various temperatures, a volumetric approach is employed. The determination of the isosteric adsorption heats of these hydrocarbons is based on single-component isotherms collected at three distinct temperatures. Additionally, the C2–4/C1 adsorption selectivities of the MOF are predicted utilizing the Ideal Adsorbed Solution Theory (IAST). To gain insights into the adsorption and separation mechanism of C2–4 hydrocarbons from CH_4_ within the Zn(bdc)(ted)_0.5_ framework, Metropolis Monte Carlo simulations are employed.

## 2. Results and Discussion

### 2.1. XRD Analysis

**Figure 1:**
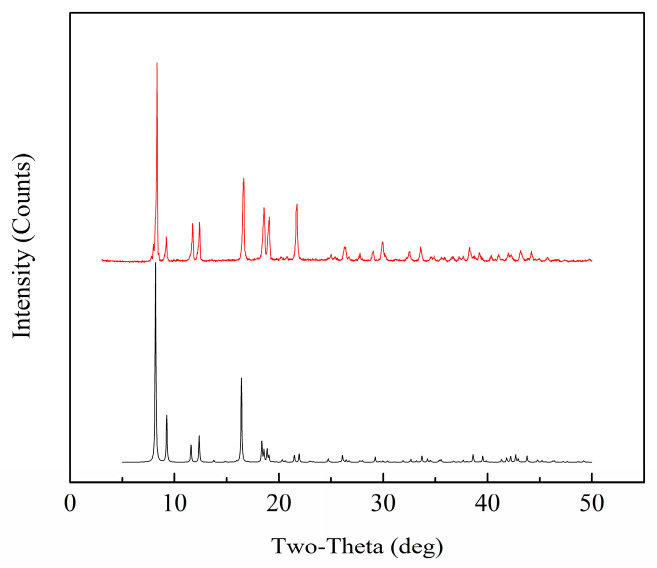
XRD patterns of the Zn(bdc)(ted)_0.5_·2DMF·0.2H_2_O (**top**) compared with the simulated pattern (**bottom**).

Figure 1 displays the X-ray diffraction pattern of the synthesized Zn(bdc)(ted)_0.5_·2DMF·0.2H_2_O sample, along with the simulated pattern for comparison. The major peaks observed in the synthesized sample closely correspond to the simulated XRD pattern, which was obtained using the Reflex module in the Materials Studio program, based on its single-crystal data [21]. Additionally, the positions of these characteristic peaks were consistent with the results reported in the literature [22]. The well-defined and resolved peaks shown in Figure 1 signify the excellent purity and crystalline nature of the sample synthesized in this study.

### 2.2. Structure Analysis

**Figure 2:**
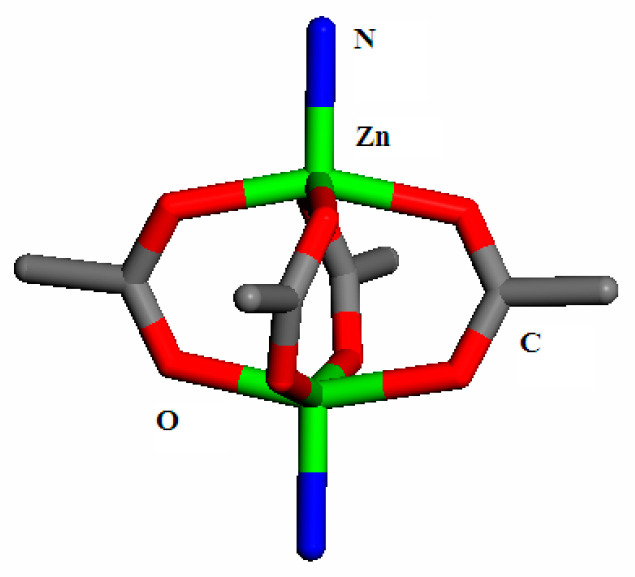
SBU unit of Zn(bdc)(ted)_0.5_, where green represents Zn, gray represents C, red represents O, and blue represents N.

Figure 2 displays the secondary building unit (SBU) of Zn(bdc)(ted)_0.5_, which is a paddle-wheel Zn_2_(COO)_4_(ted)_2_ unit. Each paddle-wheel SBU is interconnected by bdc in the two-dimensional network (xy plane), with Zn ions serving as the metal center. The apexes of Zn ions in the building units are bonded by ted molecules, forming a three-dimensional porous framework. This framework ultimately results in the formation of two interlacing channels of different sizes (7.5 × 7.5 Å and 4.8 × 3.2 Å) [23]. Zn(bdc)(ted)_0.5_ adopts the tetragonal crystal system with the space group P4/ncc [23].

### 2.3. SEM Analysis

**Figure 3:**
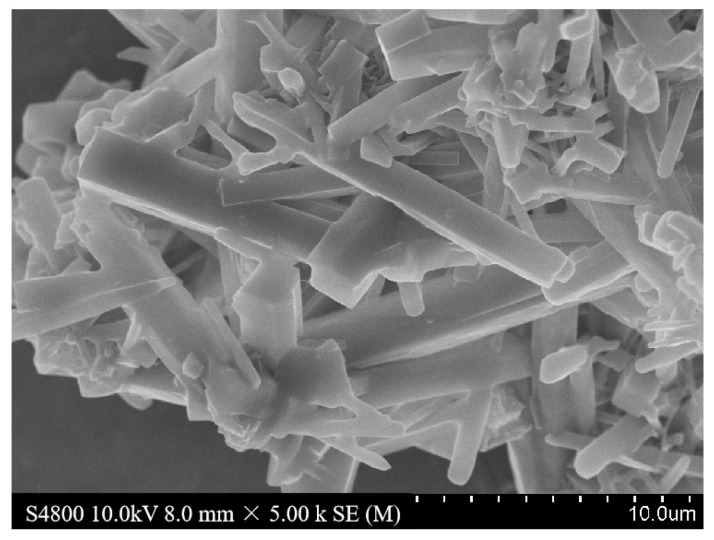
SEM image of the Zn(bdc)(ted)_0.5_·2DMF·0.2H_2_O.

Figure 3 illustrates the morphology of the synthesized Zn(bdc)(ted)_0.5_·2DMF·0.2H_2_O as observed through scanning electron microscopy. The images demonstrate that Zn(bdc)(ted)_0.5_·2DMF·0.2H_2_O exhibits well-defined crystallinity and a lump morphology [22].

### 2.4. Thermogravimetric Analysis

**Figure 4:**
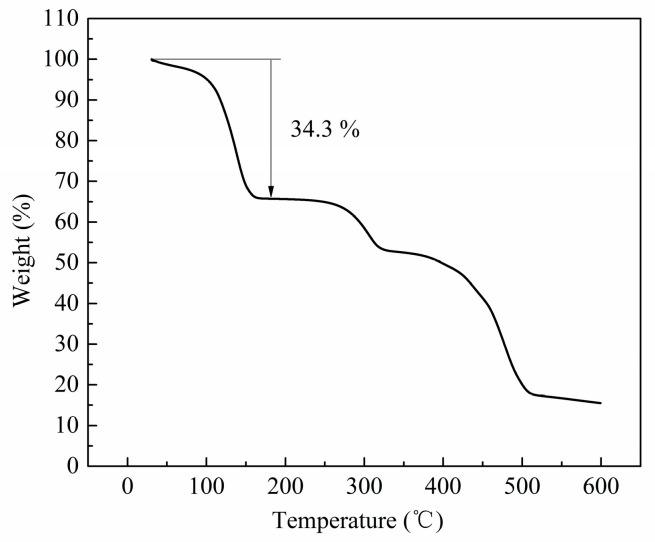
Thermogravimetric analysis of the Zn(bdc)(ted)_0.5_·2DMF·0.2H_2_O.

Figure 4 reveals the TG profile for Zn(bdc)(ted)_0.5_·2DMF·0.2H_2_O, indicating the compound’s excellent thermal stability to 260 °C. The curve reveals three well-defined weight-loss steps. The initial step, observed in the temperature range from ambient to 170 °C, corresponds to a significant weight loss of 34.3%. This weight loss is primarily attributed to the evaporation of solvent molecules (DMF and H_2_O) that are coordinated within the framework of Zn(bdc)(ted)_0.5_. The second and third steps are observed in the temperature ranges of 260–330 °C and 330–510 °C, resulting in losses of 11.6% and 17.6%, respectively, owing to the successive structural decomposition of the ted and bdc ligands.

### 2.5. Porosity Analysis

**Figure 5:**
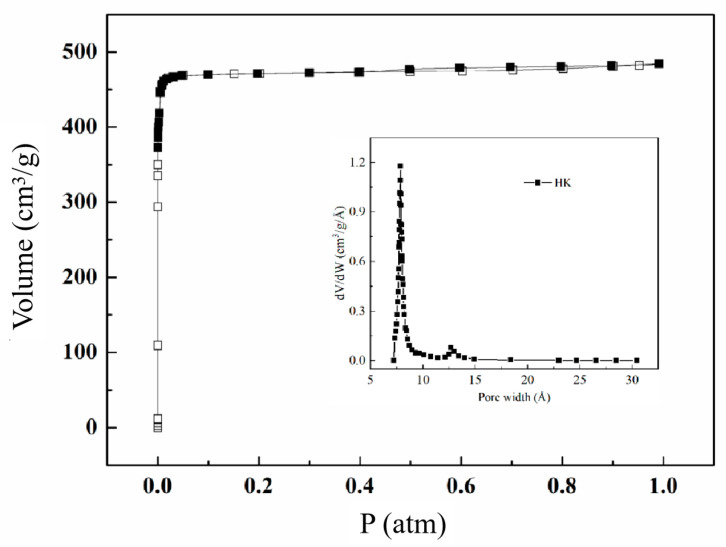
Nitrogen sorption isotherms at 77 K of the Zn(bdc)(ted)_0.5_, where the hollow square represents adsorption, and solid square represents desorption. The inset displays the pore size distribution curve that corresponds to it.

A nitrogen adsorption-desorption experiment was conducted to determine the compound’s surface area and porosity. Figure 5 presents the N_2_ sorption isotherm of Zn(bdc)(ted)_0.5_ at 77 K. The N_2_ isotherm on this compound exhibits a distinctive type-I sorption behavior, characterized by a prominent rise at low relative pressures of nitrogen, followed by a plateau. This behavior signifies the presence of abundant micropores within the adsorbent. The Langmuir and BET surface areas are determined to be 2025 and 1904 m^2^ g^−1^, respectively. The pore volume and median pore width, estimated using the Horvath-Kawazoe method, are 0.73 cm^3^ g^−1^ and 8 Å. The establishment of the permanent porosity of Zn(bdc)(ted)_0.5_ provides motivation for considering its potential application for the adsorption of light hydrocarbons.

### 2.6. Adsorption Isotherms of Hydrocarbons on the Zn(bdc)(ted)_0.5_

**Figure 6:**
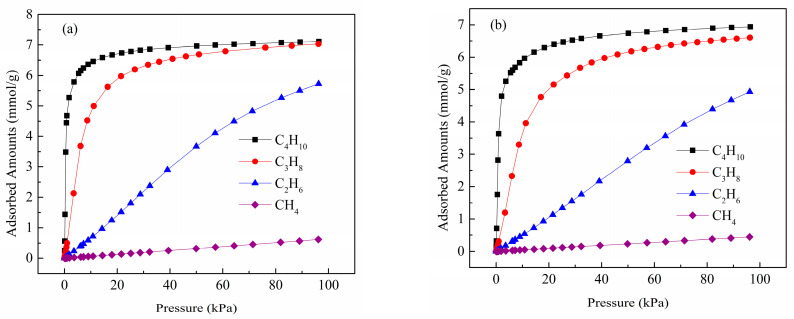
CH_4_ (purple olive), C_2_H_6_ (blue triangle), C_3_H_8_ (red circle), and C_4_H_10_ (black square) sorption isotherms of Zn(bdc)(ted)_0.5_ at (**a**) 288 K, and (**b**) 298 K.

Figure 6 represents the adsorption isotherms of light hydrocarbons (CH_4_, C_2_H_6_, C_3_H_8_, and C_4_H_10_) on the Zn(bdc)(ted)_0.5_ at temperatures of 288 K and 298 K. The figure clearly illustrates the distinct adsorption capacities of Zn(bdc)(ted)_0.5_ for CH_4_, C_2_H_6_, C_3_H_8_, and C_4_H_10_ at both 288 K and 298 K. Significantly, Zn(bdc)(ted)_0.5_ systematically adsorbs many more C2–4 hydrocarbons than C1 methane. Zn(bdc)(ted)_0.5_ demonstrates substantial adsorption capacities for C_2_H_6_, C_3_H_8_, and C_4_H_10_, reaching 5.72, 7.03, and 7.11 mmol/g at 288 K and 100 Kpa, respectively. In contrast, the uptake of CH_4_ on Zn(bdc)(ted)_0.5_ under the same conditions is significantly lower, measuring only 0.70 mmol/g. Similarly, at 298 K and 100 Kpa, Zn(bdc)(ted)_0.5_ exhibits remarkable adsorption capacities for C_2_H_6_ (4.9 mmol/g), C_3_H_8_ (6.6 mmol/g), and C_4_H_10_ (6.9 mmol/g). These uptake values significantly surpass the CH_4_ uptake (0.4 mmol/g), underscoring the exceptional suitability of Zn(bdc)(ted)_0.5_ as a highly selective material for the separation of C2–4 hydrocarbons from CH_4_ at ambient temperature.

### 2.7. Isosteric Adsorption Heats (Q_st_) of Hydrocarbons on the Zn(bdc)(ted)_0.5_

**Figure 7:**
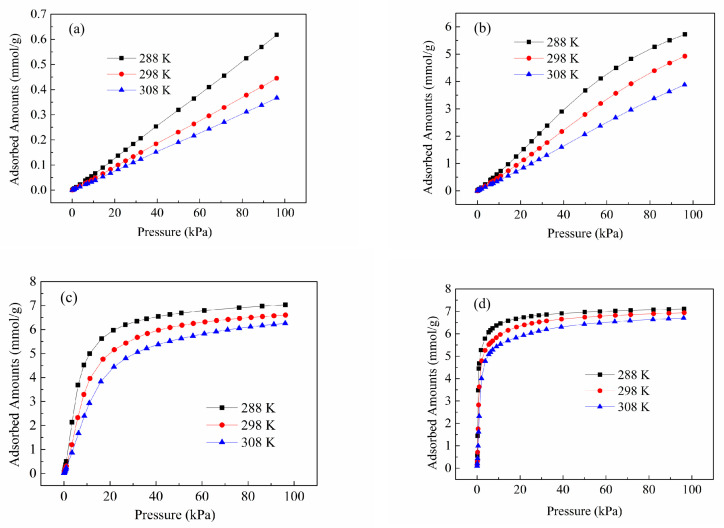
Adsorption isotherms of CH_4_ (**a**), C_2_H_6_ (**b**), C_3_H_8_ (**c**), and C_4_H_10_ (**d**) on the Zn(bdc)(ted)_0.5_ at 288–308 K.

To assess the interaction strength between the framework and gas molecules, we utilized single-component isotherms obtained at three distinct temperatures (Figure 7) to determine the isosteric adsorption heat (*Q_st_*) of hydrocarbons on the Zn(bdc)(ted)_0.5_.

Isosteric adsorption heat (*Q_st_*) can be estimated by fitting the adsorption isotherm by using the virial equation [24]:

(1) $$\ln(p)=\ln(v)+(1/T)\overset{m}{\underset{i=0}{\sum }}a_{i}v^{i}+\overset{n}{\underset{j=0}{\sum }}b_{j}v^{j}$$

where *p* (in the unit of Pa) is pressure, *v* (in the unit of mmol/g) is amount adsorbed, *T* (in the unit of K) is temperature and, $a_{i}{,\;b}_{j}$ are empirical parameters which are independent of temperature.

Adsorption isotherms obtained from different temperatures are fitted to Equation (1). Consequently, the *Q_st_* can be calculated from the following equation:

(2) $$Q_{\mathrm{st}}=-R\overset{m}{\underset{i=0}{\sum }}a_{i}v^{i}$$

where *R* is the universal gas constant (8.314 J/(K·mol)), and *Q_st_* (in the unit of J/mol) is the isosteric adsorption heat.

**Figure 8:**
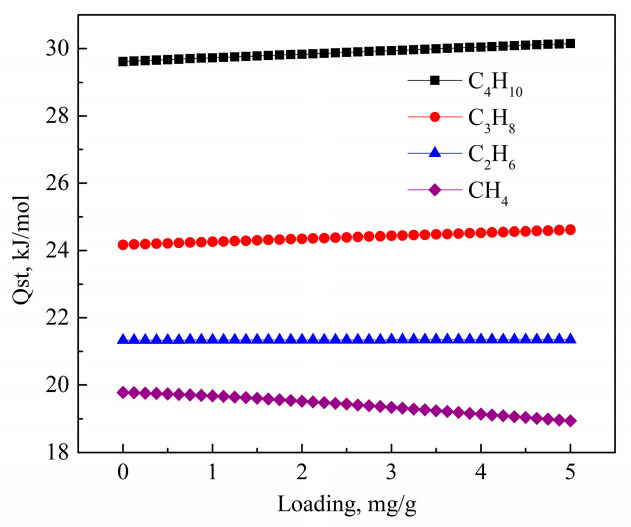
The isosteric adsorption heats of CH_4_, C_2_H_6_, C_3_H_8_, and C_4_H_10_ on Zn(bdc)(ted)_0.5_ (black square for C_4_H_10_, red circle for C_3_H_8_, blue triangle for C_2_H_6_, and purple olive for CH_4_).

Figure 8 displays the *Q_st_* of CH_4_, C_2_H_6_, C_3_H_8_, and C_4_H_10_ on Zn(bdc)(ted)_0.5_. The *Q_st_* values of C_2_H_6_, C_3_H_8_, and C_4_H_10_ on the Zn(bdc)(ted)_0.5_ are higher than the *Q_st_* of CH_4_, indicating a stronger interaction between the C_2_H_6_, C_3_H_8_, C_4_H_10_, and the Zn(bdc)(ted)_0.5_ surfaces. Specifically, the *Q_st_* values of CH_4_, C_2_H_6_, C_3_H_8_, and C_4_H_10_ are around 19.8, 21.3, 24.2, and 29.6 kJ mol^−1^, causing the preferential adsorption of C_2_H_6_, C_3_H_8_, and C_4_H_10_ over CH_4_.

### 2.8. Ideal Adsorbed Solution Theory Selectivity

Materials with the potential for CH_4_ purification require not only high C2–4 uptake and low CH_4_ uptake but also remarkable selectivity for C2–4/CH_4_. The above results reveal that Zn(bdc)(ted)_0.5_ possesses a high C2–4 uptake and a low CH_4_ uptake, which prompts further investigation into the gas selectivity for C2–4/CH_4_. The IAST model is commonly employed for predicting the adsorption selectivity of binary gas mixtures using experimental data from pure gas isotherms. In this study, to characterize the adsorption behavior of hydrocarbons on Zn(bdc)(ted)_0.5_ and perform the IAST calculation, the dual-site Langmuir–Freundlich (DSLF) equation is applied to correlate with the experimental isotherm data of the hydrocarbons. The DSLF model can be mathematically expressed as:

(3) $$q=q_{1}^{\max}\times \frac{b_{1}p^{{1/n}_{1}}}{{1+b}_{1}p^{{1/n}_{1}}}{+q}_{2}^{\max}\times \frac{b_{2}p^{{1/n}_{2}}}{{1+b}_{2}p^{{1/n}_{2}}}$$

where *q* (in the unit of mmol/g) represents the adsorbed uptakes per mass of adsorbent, *p* (in the unit of kPa) represents the pressure of the bulk gas in equilibrium with the adsorbed phase, and $q_{1}^{\max}$ and $q_{2}^{\max}$ (in the unit of mmol/g) denote the saturated adsorption capacities of sites 1 and 2, respectively. The parameters *b*_1_ and *b*_2_ (in the unit of kPa^−1^) indicate the affinity coefficients of sites 1 and 2, and *n*_1_ and *n*_2_ represent the deviations from an ideal homogeneous surface.

**Figure 9:**
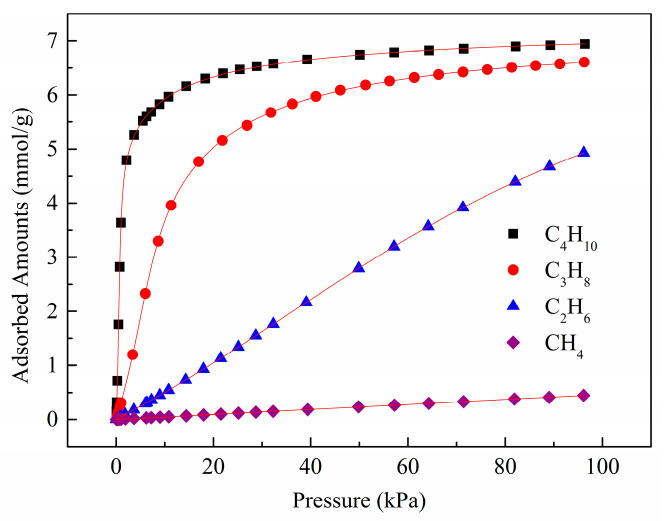
Experimental and fitted isotherms for CH_4_, C_2_H_6_, C_3_H_8_, and C_4_H_10_ at 298 K (black square for C_4_H_10_, red circle for C_3_H_8_, blue triangle for C_2_H_6_, and purple olive for CH_4_).

Figure 9 presents a comparison between the DSLF model fittings and the experimental isotherm data. The corresponding fitting parameters and the regression coefficients are listed in Table 1. The high regression coefficient *R*^2^ (up to 0.9999) indicates a good fit between the DSLF model and the single-component experimental isotherms. Therefore, the IAST model is implemented using pure gas adsorption fitted parameters.

**Figure 10:**
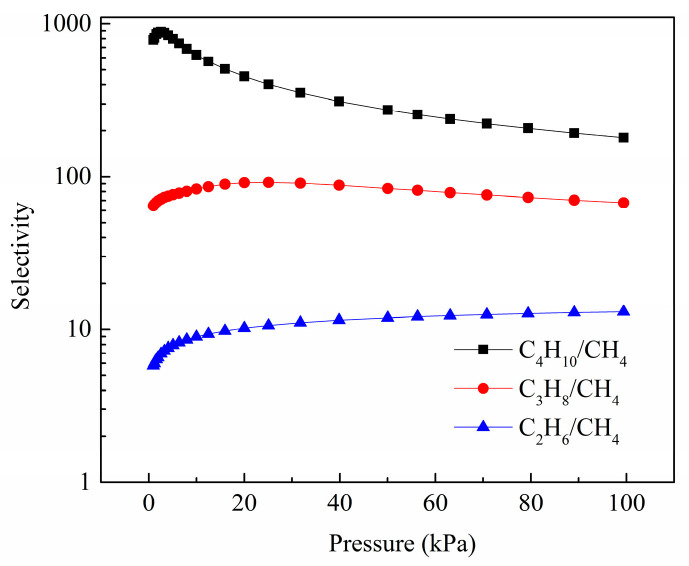
IAST-predicted equimolar gas mixture adsorption selectivities for Zn(bdc)(ted)_0.5_ at 298 K (black square for C_4_H_10_/CH_4_, red circle for C_3_H_8_/CH_4_, and blue triangle for C_2_H_6_/CH_4_).

The IAST approach, originally developed by Myers and Praunitz [25], has been extensively employed to predict adsorption selectivity based on the isotherms of the pure components. Figure 10 presents the IAST calculations of C_4_H_10_/CH_4_, C_3_H_8_/CH_4_, and C_2_H_6_/CH_4_ selectivities for an equimolar binary mixture maintained at isothermal conditions at 298 K. In general, the adsorption selectivities follow the order C_4_H_10_/CH_4_ > C_3_H_8_/CH_4_ > C_2_H_6_/CH_4_. This observation can be attributed to the enhanced interaction between the material and C2–4 in comparison to CH_4_, suggesting a stronger affinity of the material towards C2–4. At 100 kPa, the C_4_H_10_/CH_4_ selectivity is up to 180, the C_3_H_8_/CH_4_ selectivity is 67, and the C_2_H_6_/CH_4_ selectivity is 13. In conclusion, Zn(bdc)(ted)_0.5_ exhibits a significant adsorption capacity for C2–4 hydrocarbons and demonstrates a high C2–4/CH_4_ adsorption selectivity. These findings underscore the potential of this material as a highly promising candidate for effectively separating C2–4/CH_4_ mixtures.

Table 2 lists the adsorption separation performance of light hydrocarbons for several benchmark MOFs at 298 K and 100 kPa. Notably, Zn(bdc)(ted)_0.5_ demonstrates a high ethane adsorption capacity, ranking third among the listed adsorbents and reaching an excellent level. Conversely, the methane adsorption capacity is extremely low, resulting in a remarkably high selectivity for C_2_H_6_/CH_4_ separation. To the best of our knowledge, the ethane storage capacity of Zn(bdc)(ted)_0.5_ surpasses that of CTGU-15 (2.13 mmol/g) [18], PCN-224 (2.93 mmol/g) [20], MIL-100 (Fe) (2.22 mmol/g) [17], MIL-142A (3.82 mmol/g) [26], UIO-67 (4.26 mmol/g) [27], Iso-MOF-1 (3.19 mmol/g) [28], InOF-1 (4.14 mmol/g) [19], JIU-Liu22 (3.30 mmol/g) [29], JIU-Liu37 (4.42 mmol/g) [30], and FJI-C1 (3.93 mmol/g) [31]. It is inferior to the corresponding values of JIU-Liu38 (4.96 mmol/g) [30] and and FJI-H23 (6.28 mmol/g) [32]. Overall, based on the data in Table 2, Zn(bdc)(ted)_0.5_ exhibits competitive ethane uptake and exceptional C_2_H_6_/CH_4_ adsorption selectivity, positioning it as a promising candidate for C_2_H_6_/CH_4_ separation.

### 2.9. Simulation Results

**Figure 11:**
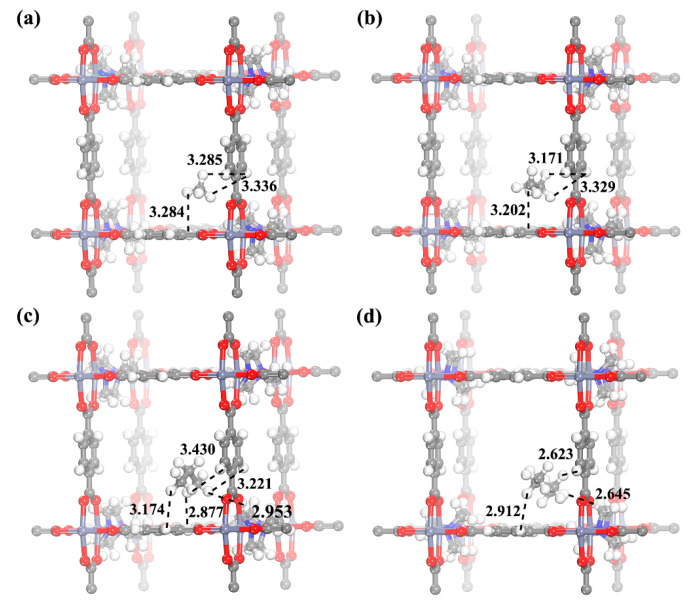
The calculated preferential adsorption site for a single (**a**) CH_4_, (**b**) C_2_H_6_, (**c**) C_3_H_8_ and (**d**) C_4_H_10_ in Zn(bdc)(ted)_0.5_.

Figure 11 shows the calculated preferential adsorption sites in the adsorbent for a single adsorbate molecule, with the distance between the H atoms of the adsorbate and the H atoms of bdc indicated. The calculation results indicate that both C1 and C2 molecules tend to be adsorbed in the region sandwiched by two adjacent bdc ligands. Unlike C1 and C2 molecules, the ted ligand also exhibits a certain attraction towards C3 and C4 molecules, which tend to be adsorbed at the vertex of the rectangular cage. Therefore, it can be concluded that the interaction between C1/C2 molecules and the adsorbent is mainly attributed to the dispersion force between bdc and the hydrogen atoms from the adsorbate molecules. For C3 and C4 molecules, in addition to the interaction with bdc ligands, the methylene group on the ted ligand also provides a certain amount of dispersion force.

As the carbon chain length increases, the shortest distance between the adsorbate and the adsorbent gradually decreases from 3.285 Å for CH_4_ to 3.171 Å for C_2_H_6_, 2.877 Å for C_3_H_8_, and 2.623 Å for C_4_H_10_, suggesting an increasing dispersion force. This is because the growing carbon chain makes the polarity of the adsorbate molecules gradually larger, resulting in a larger instantaneous dipole moment during adsorption. Compared with the bdc ligand, the ted ligand is less polar, so the interaction between the ted ligand and C1/C2 molecules is weaker. As the polarity of the adsorbate molecules increases, the ted ligand also participates in the interaction with the adsorbate molecules (the distances for C3 of 2.953 Å and C4 of 2.645 Å are both smaller than the shortest distance between the C3 or C4 molecule and the framework), so the selectivity of C3/C1 and C4/C1 is greater than that of C2/C1.

Additionally, it is worth noting that the closest H atom in C_3_H_8_ to the bdc ligand comes from the methyl group, while in C_4_H_10_, the closest H atom to the ligand comes from the methylene group. With the synergistic effect of adjacent bdc ligands and ted ligands, more abundant H atoms and a longer carbon chain from C_4_H_10_ prompt the C4 molecule to fully interact with the skeleton.

**Figure 12:**
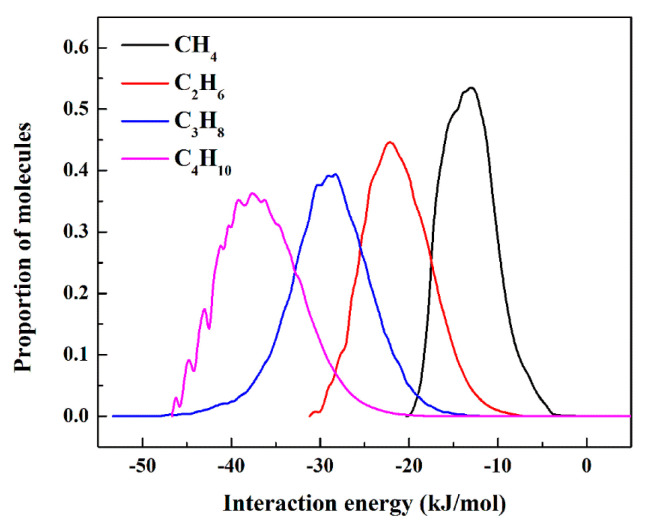
Interaction energy histogram for C1−C4 with Zn(bdc)(ted)_0.5_.

Figure 12 shows the interaction energy histograms for different adsorbate molecules and the adsorbent. From Figure 10, it can be seen that the order of adsorption energies between C1–C4 molecules and the adsorbent is C_4_H_10_ (−37.5 kJ/mol) > C_3_H_8_ (−28.7 kJ/mol) > C_2_H_6_ (−22.1 kJ/mol) > CH_4_ (−13.0 kJ/mol). As the length of the carbon chain increases, the height of the peaks in the energy curve gradually decreases, and the width of the peaks gradually increases. On the one hand, the number of primary adsorption sites between the adsorbate and the adsorbent was changed from a single ligand to two types of ligands. On the other hand, the peak of the C4 molecule is wider and higher than that of the other molecules due to the participation of rich methylene groups in the adsorbate-adsorbent interaction, which agrees with the result from Figure 9.

## 3. Materials and Methods

### 3.1. Synthesis

The chemicals utilized in this study were purchased from reputable commercial sources and used as received without undergoing any additional purification steps. The starting materials utilized include zinc nitrate hexahydrate [Zn(NO_3_)_2_·6H_2_O, Alfa, 98%], terephthalic acid (Alfa, 98%), 1,4-diazabicyclo[2.2.2]octane (TCI, 98%), N,N-dimethylformamide (DMF, Guanghua Chemical Co., Ltd., Guangzhou, China, 99.8%), N_2_, CH_4_, C_2_H_6_, C_3_H_8_, and C_4_H_10_ (99.999%, Guangdong Huate Gas Co., Ltd., Foshan, China).

The synthesis of Zn(bdc)(ted)_0.5_·2DMF·0.2H_2_O was carried out using the following procedure: a mixture of zinc(II) nitrate hexahydrate [Zn(NO_3_)_2_·6H_2_O, 0.597 mmol, 156 mg], terephthalic acid (H_2_bdc, 0.614 mmol, 102 mg), and 1,4-diazabicyclo[2.2.2]octane (ted, 0.321 mmol, 36 mg) was dissolved in 15 mL of dimethylformamide in a 20 mL glass vial. The solution was then subjected to sonication for a duration of 10 min and subsequently heated at 120 °C for 48 h. After the completion of the reaction, the resulting crystals were obtained with a yield of 78%. The crystals were then filtered and subjected to three consecutive washes using 10 mL of DMF each time. The final product weighed 200 mg.

### 3.2. Characterization

X-ray diffraction patterns were obtained using a Rigaku D/M-2200T automated diffraction system (Ultima IV) equipped with Cu-Kα radiation (λ = 1.5406 Å). The measurements were carried out at room temperature, employing a 2θ range of 3–50°, a scan speed of 2°/min, and an operating voltage of 40 kV and current of 40 mA. Scanning electron microscopy (SEM) analysis was performed using a Hitachi S-4800 instrument after the deposition of a thin layer of gold. Thermogravimetric analyses were performed using a TA Q5000 analyzer, which ramped the temperature from ambient to 600 °C at a heating rate of 10 °C/min under a continuous nitrogen gas atmosphere. Nitrogen adsorption isotherms at 77 K were collected using a Micromeritics ASAP 2020 instrument. Prior to the measurements, the sample underwent an initial outgassing process overnight under vacuum at 393 K.

### 3.3. Adsorption of Light Hydrocarbons

Gas sorption experiments for CH_4_, C_2_H_6_, C_3_H_8_, and C_4_H_10_ at various temperatures (288 K, 298 K, and 308 K) were conducted using high-resolution Micromeritics 3-Flex adsorption equipment. The gases employed in the experiments were of ultra-high purity (99.999%). Prior to the measurements, the samples underwent an initial outgassing process at 393 K under vacuum overnight. Outgassing samples of approximately 100 mg were used for gas sorption measurements.

### 3.4. Simulation

In order to investigate the adsorption and separation mechanism of C2–4 hydrocarbons over CH_4_ in Zn(bdc)(ted)_0.5_, the Metropolis Monte Carlo simulation by Materials Studio 7.0 [33] was employed to mimic the adsorption behavior of the adsorbate in the MOF structure. The initial structure of Zn(bdc)(ted)_0.5_ obtained from the Cambridge Crystallographic Data Centre [21] was optimized using the CASTEP module with convergence criteria set at 2 × 10^−5^ ev for energy, 0.1 ev/Å for force, 0.1 GPa for stress, and 2 × 10^−3^ Å for displacement. The optimization employed the GGA/PBE basis was set with the Grimme method for dispersion correction, a commonly used approach for crystal structure optimization. The energy cutoff was set at 300.0 ev and the ultrasoft pseudopotential was applied. After optimization, the sorption module was utilized to explore the gas adsorption behavior within a 2 × 2 × 2 supercell of the optimized Zn(bdc)(ted)_0.5_ structure. During the Metropolis Monte Carlo simulation, various moves, such as insertion, transformation, deletion, rotation, and regrowth, were employed to mimic the adsorption behavior of hydrocarbons. The simulation cutoff distance was set at 12.0 Å. The interactions between adsorbate-adsorbent and adsorbate-adsorbate were described using the Lennard-Jones 12-6 potential [34]. Force field parameters for each atom were obtained from the universal force field [35]. To determine the preferred adsorption site of the Zn(bdc)(ted)_0.5_ structure for hydrocarbon molecules, a single adsorbate molecule was randomly inserted into the MOF structure using the location task in the Sorption module.

## 4. Conclusions

This study synthesized a microporous metal-organic framework Zn(bdc)(ted)_0.5_ for adsorbing and separating light hydrocarbons (CH_4_, C_2_H_6_, C_3_H_8_, and C_4_H_10_). The material exhibits a BET surface area of 1904 m^2^/g and a pore volume of 0.73 cm^3^/g. The resulting Zn(bdc)(ted)_0.5_ exhibits good separation performance for the recovery of C_2_H_6_, C_3_H_8_, and C_4_H_10_ from CH_4_. Notably, the material shows a significantly high C_2_H_6_ adsorption capacity of 4.9 mmol/g (ranking among the top reported MOFs) and a relatively low CH_4_ adsorption capacity of 0.4 mmol/g at 298 K and 100 kPa. The isosteric adsorption heats of C_2_H_6_, C_3_H_8_, and C_4_H_10_ on the Zn(bdc)(ted)_0.5_ are higher than that of CH_4_, indicating a stronger interaction between C_2_H_6_, C_3_H_8_, and C_4_H_10_ molecules and Zn(bdc)(ted)_0.5_ and demonstrating the results of single-component isotherms and selectivities. At 100 kPa, the C_4_H_10_/CH_4_ selectivity is up to 180, the C_3_H_8_/CH_4_ selectivity is 67, and the C_2_H_6_/CH_4_ selectivity is 13. The molecular simulation results demonstrate that Zn(bdc)(ted)_0.5_ exhibits a preference for adsorbing hydrocarbon molecules with a higher abundance of C-H bonds and larger polarizability. This preference leads to the generation of stronger dispersion forces through the induced polarization effect between the adsorbent and the adsorbate. In conclusion, Zn(bdc)(ted)_0.5_ has significant potential for application in the recovery of C_2–4_ from natural gas.

## Figures and Tables

**Table 1:** Equation coefficients for the DSLF isotherm model.

Adsorbates	$q_{1}^{\max}$ (mmol/g)	*b*_1_ (kPa^−1^)	*n*_1_	$q_{2}^{\max}$ (mmol/g)	*b*_2_ (kPa^−1^)	*n*_2_	*R* ^2^
CH_4_	16.5729	1.359 × 10^−4^	0.9918	16.573	1.0359 × 10^−4^	0.9918	0.9999
C_2_H_6_	7.07662	0.0022	0.8282	7.0766	0.0022	0.8282	0.9999
C_3_H_8_	1.50532	0.0016	0.2973	5.5847	0.0588	0.8818	0.9999
C_4_H_10_	3.50191	3.031	0.3748	3.8683	0.3413	1.4425	0.9999

**Table 2:** Comparison of adsorption separation performance of light hydrocarbons among several benchmark MOFs at 298 K and 100 kPa.

Adsorbents	BET	C_2_H_6_	CH_4_	C_2_H_6_/CH_4_	References
	(m^2^ g^−1^)	(mmol/g)	(mmol/g)	Selectivity	
CTGU-15	3163.7	2.13	0.4	5.2	[18]
PCN-224	2704	2.93	0.48	12	[20]
MIL-100(Fe)	2482	2.22	0.36	6	[17]
MIL-142A	1424.7	3.82	0.54	14.5	[26]
UIO-67	2590.6	4.26	0.56	8.1	[27]
Iso-MOF-1	3211	3.19	0.38	-	[28]
InOF-1	982	4.14	0.64	17	[19]
JLU-Liu22	1487	3.3	0.71	14.4	[29]
JLU-Liu37	1795	4.42	0.45	11	[30]
JLU-Liu38	1784	4.96	0.48	15	[30]
FJI-C1	1726.3	3.93	0.45	24	[31]
FJI-H23	3740.4	6.28	0.67	14.7	[32]
Zn(bdc)(ted)_0.5_	1904	4.9	0.4	13	This work

## Data Availability

Data is contained within the article.
